# Mr. Scott's Case of Death after the Pumping of Rum

**Published:** 1809-07

**Authors:** James Scott

**Affiliations:** Surgeon. H. M. S. Euryalus, Sheerness


### *To the Editors of the Medical and Phyſical Journal.*

GENTLEMEN,

I AM much obliged by your publishing my case of Hydrothorax, and with pleasure hasten to answer your questions relative to the time that elapsed between the patient's death and the inspection of his body, and "whether there was any reason to suspect transudation after death."

My only object in trespassing on the pages of your valuable Journal, is to arrive at the truth, and, if I can, to benefit science; I am therefore willing, as far as I am able, to give the fullest information on any circumstances that have been inadvertently omitted in my paper. I am open to conviction; and being a very young practitioner, return you my best thanks for the liberal manner in which your questions are proposed.

In the Navy we have frequent cause to lament the superstitious aversion which sailors have to dead bodies being opened; and I am sorry to add, that in most of his Majesty's ships this abhorrence of what ought to be considered a sacred duty of the surgeon, pervades all ranks. It has, however, been my good fortune to serve with officers of liberal understanding; and in the few mortal cases that have occurred in my practice, I have uniformly obtained the consent and even approbation of the crew, by fairly stating the object of my research, and allowing such of them as chose it, to witness the dissection.

At the death of the patient whose case is under our present consideration, I was most anxious for immediate inspection of the body; but owing to the Captain's absence, could not accomplish my wish till the next morning; so that nearly fourteen hours elapsed between the dissolution and the opening of the body. With respect to your second question, I will candidly confess that I did not once think of such a circumstance as "transudation after death;" nor can I now reconcile it with my ideas of organized bodies.

The accumulation of fluids in the thorax, in the ventricles of the brain, and in all other cavities of the body, I conceive can only take place while the vital principle is resident in the frame, or not after death till the corpse has begun to turn putrid; and although in those complaints which have been denominated "malignant," appearances of of putrefaction often manifest themselves almost immediately after death, it more commonly happens (unless the weather be excessively warm) that no perceptible change takes place for several days; and sometimes interment is not necessary, on account of the body's corruption, for a fortnight even, in a temperate climate. Just before death we frequently find a momentary increased action of the circulating and absorbing systems, followed by an involuntary discharge of bilious and mucous excrement, flux of tears and saliva, with cold, clammy, perspirable matter left on the surface of the body, all consequences of preceding laborious action, which would appear to be a last friendly effort of the vis medicatrix naturæ. I must therefore still believe, that in my patient the effusion took place before death. And here I would beg leave to make a quotation from a work highly and deservedly estimated, which will shew I am not singular in the belief that Hydrothorax may exist, without any of the symptoms which have been considered diagnostic. After a particular enumeration of the usual symptoms of thoracic dropsy, we find the following passages.

"That these symptoms are common attendants of this disease (Hydrothorax) is undeniable; and they are certainly the best characteristics of this affection with which we are yet acquainted: but it must be allowed that they are present in some cases where there is no water in the breast; and that in other instances where the disease exists, they are either altogether wanting, or occur only in a very slight degree. Certain diagnostics therefore of this disease still remain to be discovered."*

But notwithstanding my opinion must be considerably strengthened by the above quotation, it may still be erroneous; and as I have delivered my sentiments with candour, and, I hope, with respectful freedom, I trust you, or some of your Correspondents, will favour me, and the cause of science, by a reciprocation of ideas, which cannot but tend to the improvement of our art.

With your permission, I will state a case of gangrenous pylorus, which I believe was occasioned by spirituous potation. On the 14th day of July, 1808, in the afternoon, while we were at anchor in the roads of New Providence, Cornelius Collopy, seaman, ætat. 23, of a full habit, and who

* Edinburgh Practice of Physic, &c. Vol. ii. p. 474.

who had enjoyed excellent health, with the exception of an occasional slight dyspnœa, was employed in pumping new rum from large casks into smaller ones; and although much care was taken to prevent his drinking any liquor, he appeared in the evening to be very much intoxicated. Two other men, who were engaged in the same duty, were also drunk; but the officer who attended to see the spirits drawn off, declared they did not drink any, and attributed their drunkenness to the "fumes" or evaporation of the rum, which certainly was very strong. At eight o'clock, P. M. when Collopy's ebriety was first discovered, he was evidently in a state of the utmost danger, having entirely lost the power of speech and voluntary motion. The countenance, notwithstanding, was but slightly flushed; and although he had a little stertorous breathing, there did not appear to be much determination to the head. The region of the stomach was distended, seemingly with flatus, and just below the scrobiculus cordis I observed a circumscribed tumour, very compressible, nearly the size of a hen's egg. Under such circumstances, bleeding did not seem advisable, because of the extreme debility which must necessarily succeed the vast excitement of the spirits; and as the only chance of saving the patient appeared to depend on the contents of his stomach being speedily evacuated, fifteen grains of pure sulphate of zinc were immediately given, and followed by frequent draughts of tepid water, which, it was hoped, would accelerate and assist the action of the white vitriol. At nine, P. M. very little vomiting had been induced, but the patient, though quite unconscious, voided a great quantity of limpid urine, and had an involuntary motion per anum. The dose of sulphate of zinc was therefore repeated, with two oz. of olive oil, and some lukewarm water, as before. At ten, P. M. he continued in a state of perfect insensibility; and notwithstanding thirty grains of sulphate of zinc in solution had been injected into the stomach, its contents were obstinately retained. The heart seemed to contract very irregularly, and at times to intermit, which, with the inefficacy of the emetic, pretty clearly indicated the destruction of irritability in the stomach; and a little before twelve, P. M. he ceased to exist.

### Appearances on Dissection.

As this man's death was so sudden, and altogether unexpected by the ship's crew, I judged it my duty to explain the cause of his dissolution, and more particularly so, as opening opening the body in their presence would afford me an opportunity to point out to the sailors, the dreadful effects of intoxication. The surface of the brain exhibited no signs of inflammation, and only a very small quantity of fluid was found in the ventricles. The cerebellum appeared to be perfectly sound. The thoracic viscera had no marks of disease, except rather a greater proportion of fat than is usually found attached to the heart, and which I suppose occasioned the dyspnœa mentioned above. On laying open the abdomen, the stomach appeared prodigiously enlarged, and had occasioned the compressible tumour at the scrobiculus cordis. The cardia had no uncommon appearance, nor did the peritoneal coat indicate any of the effects of inflammation; but on opening the stomach, I observed the tunica glandulosa, or villosa, of a florid colour, and in some parts with every appearance of the most violent inflammatory action. The pylorus was preternaturally thick, and had a gangrenous triangular spot on its upper part, about one inch and a half in perpendicular extent, and with a base about an inch. The stomach contained fully a pint of a greyish fluid, (in which the presence of spirituous liquor could not be detected) mixed with pieces of undigested bread and fruit, which seemed to have undergone a process of fermentation, during which, much gas had been evolved, and the flatulent distension of the stomach produced. Whether the "fumes" of liquor could have caused such effects, I will not "assume the province of determining," but I rather think the spirit was bodily present.

I am, &c.

*H. M. S. Euryalus, Sheerness,* JAMES SCOTT, Surgeon.

*June* 4, 1809.

---

EDITORS' REMARK.

Transudation after death is by no means a common case, and depends on the manner in which the subject died. In animals slaughtered in the usual way, we believe it has never been recorded. Mr. Scott's remarks on the appearances immediately before death, and which cease with death, show his habits of observation, and convince us that by his diligence and opportunities, some light may be thrown on a most important branch of pathology, namely, a distinction between those appearances which may be considered as the *causa mortis*, and such as may have existed without materially injuring the functions of life, or have occurred in *articulo mortis*, or even after apparent death.

The

The appearances marked by Mr. Scott as often preceding death, in what are called putrid diseases, cease, as he observes, with death, and the body keeps longer than one which died suddenly and in high health. Another remark may be made, that after death, or more properly after respiration has ceased, and the body has become cold, it will recover its warmth, and the cheeks regain their colour. If at this time a vein is opened, the blood will flow, and afterwards coagulate, and the limbs stiffen. All this arises from a process which takes place after the other actions by which life is supported have ceased. But they must be the effect of life remaining in certain parts. Accordingly we find, that under certain modes of dying these actions subsequent on the cessation of respiration never take place; that no heat returns; the blood is not thrown by the contraction of the arteries into the veins; the blood never coagulates, the body never stiffens, and putrefaction takes place early. These cases usually happen in hot seasons, after violent deaths or very short illnesses; and in some such instances the stomach is sometimes found digested.

